# Consenting Operative Orthopaedic Trauma Patients: Challenges and Solutions

**DOI:** 10.1155/2014/354239

**Published:** 2014-02-06

**Authors:** Amin Kheiran, Purnajyoti Banerjee, Philip Stott

**Affiliations:** Department of Trauma & Orthopaedic Surgery, RSCH, Brighton Sussex University Hospitals, Brighton BN2 5BE, UK

## Abstract

Guidelines exist to obtain informed consent before any operative procedure. We completed an audit cycle starting with retrospective review of 50 orthopaedic trauma procedures (Phase 1 over three months to determine the quality of consenting documentation). The results were conveyed and adequate training of the staff was arranged according to guidelines from BOA, DoH, and GMC. Compliance in filling consent forms was then prospectively assessed on 50 consecutive trauma surgeries over further three months (Phase 2). Use of abbreviations was significantly reduced (*P* = 0.03) in Phase 2 (none) compared to 10 (20%) in Phase 1 with odds ratio of 0.04. Initially, allocation of patient's copy was dispensed in three (6% in Phase 1) cases compared to 100% in Phase 2, when appropriate. Senior doctors (registrars or consultant) filled most consent forms. However, 7 (14%) consent forms in Phase 1 and eleven (22%) in Phase 2 were signed by Core Surgical Trainees year 2, which reflects the difference in seniority amongst junior doctors. The requirement for blood transfusion was addressed in 40% of cases where relevant and 100% cases in Phase 2. Consenting patients for trauma surgery improved in Phase 2. Regular audit is essential to maintain expected national standards.

## 1. Introduction

Health professionals have a legal and ethical obligation to obtain valid informed consent before any procedure to be performed. Consent is as fundamental as any other basic principle on which surgical practice relies, and its use in patient care is a clinical skill [1]. Guidelines have been developed by professional bodies highlighting the importance of the principles and the process of seeking consent [2–4]. The importance of establishing informed consent form is to reflect the requirement to ensure that essential and sufficient information has been imparted between patient and health professional (surgeon) [4]. Consent is a two-way process. The health professional should know the patient's history and problems. The patient must be given time to ask relevant questions. The health professional must check understanding and offer alternative treatments. They must explain relevant risks and benefits. This combination will ensure that the correct procedure is being performed on the correct patient [5]. The requirement for consent has now explicitly been extended to the disposal of human tissue and the management of personal data. Although these extensions are not a central issue for surgeons,they obviously include massive implications during the consent process. Hence, the other members of the involved team will be reassured that the patient's consent has been appropriately given, preserving their autonomy [1]. Use of abbreviations, acronyms, and symbols is proliferating. They convey a different meaning in a different context, which causes misunderstanding, decreasing the effectiveness of communication among caregivers, and may lead to unsafe practice [6, 7]. There are indications from recent judgments by which inadequate documentation has implications in medicolegal cases [2, 8]. General Medical Council Guidelines published in 1998 stress the need to obtain consent as otherwise there is a risk of litigation.

Current GMC guidance to consent indicates that if the delegated junior levels (Core Trainee) are subject to gain informed valid consent, they must have sufficient knowledge of the proposed treatment (e.g., hip hemiarthroplasty procedure) or investigation [2]. Therefore, they must be suitably trained and supervised [2].

The purpose of this study was to monitor compliance of the consent form completion amongst patients undergoing orthopaedic trauma surgery (who sustained severe fracture pattern requiring operative-based treatment) with existing approved consent guidelines. This investigation was aimed to include quality of record keeping in the consent form and deficiencies in documentation. Therefore, we intended to recommend changes, to review potential improvement that could be established, and to increase awareness ensuring good practice in consenting and record keeping across the Department and the Trust.

## 2. Patients and Methods

We have reviewed case notes of trauma patients who had undergone operative procedures over two separate phases.

**Phase 1**

A retrospective review of fifty surgical consent forms undergoing orthopaedic procedures were undertaken from November 2011 to January 2012 at a level 1 major trauma center. Two separate weeks were randomly chosen in the three-month period in order to minimise bias. The inclusion criteria were all patients who were able to consent voluntarily for an orthopaedic procedure. We excluded any patients who were unable to consent due to acute or chronic deficiency of mental capacity to consent for surgery. Furthermore, all complex polytrauma patients who had multiple surgeries by different surgical specialties were also excluded. Data was collected by using project-specific questionnaire designated for the quality of the consent form documentation developed by Clinical Effectiveness Support Unit (CESU) in the National Health Service (NHS) Trust.

A database was created to collect the data systematically by one of the authors (AK) who was not directly involved in completing these consenting forms. The outcome included

- evaluating the adequacy of medical data for each procedure,
- use of abbreviation,
- type of anaesthesia, for example, general/regional or local anaesthesia,
- documentation of relevant procedure for treatment,
- additional record for blood transfusion and/or extra-procedures if required,
- grade of delegated health professional gaining consent,
- confirmation of consent form,
- allocation of patient's copy.

The collected data for retrospective study was presented in the departmental clinical governance meeting and subsequently circulated to the NHS Audit Department. The results were circulated amongst the member of staff and necessary training sessions were arranged to ensure adherence to national-based issued guidance in consenting practice for trauma patients undergoing surgery was improved and maintained.

**Phase 2**

A prospective review of fifty further consent forms was undertaken in order to assess the impact of our training subsequent to Phase 1. The case notes of patients consenting for trauma surgery in the orthopaedic unit in two random weeks between May to July 2012 were chosen. The staff were unaware of this audit and the data was collected by one of the authors (AK) not involved in the consent taking in any case. The outcome measures were same as in Phase 1. Full approval from the Audit Department was obtained prior to this study.

## 3. Results

Retrospectively, in Phase 1, fifty case notes were reviewed; in 6% (*n* = 3) of these, there were no consent form attached to the case note who had surgical operation. All the patients had consent forms available for review in Phase 2 (*n* = 50).

**Figure 1:**
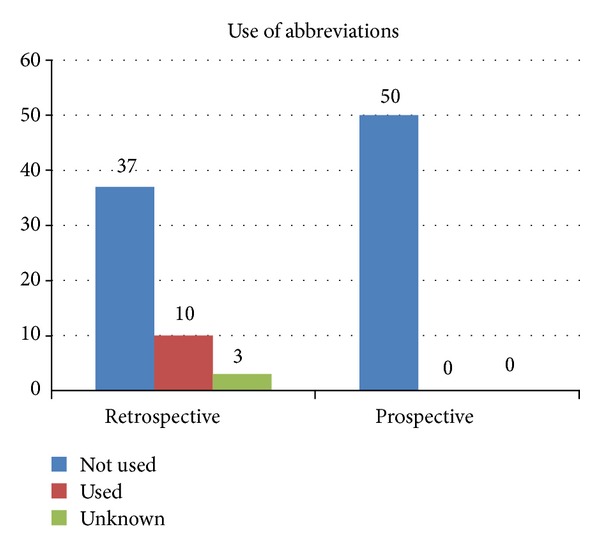
Use of “Abbreviation(s)” in the consent form documentations.

Use of abbreviations was significantly reduced (*P* = 0.03) in Phase 2 (none) compared to 10 (20%) in Phase 1 with odds ratio of 0.04 (Figure 1). All consent forms described procedures using plain language.

**Figure 2:**
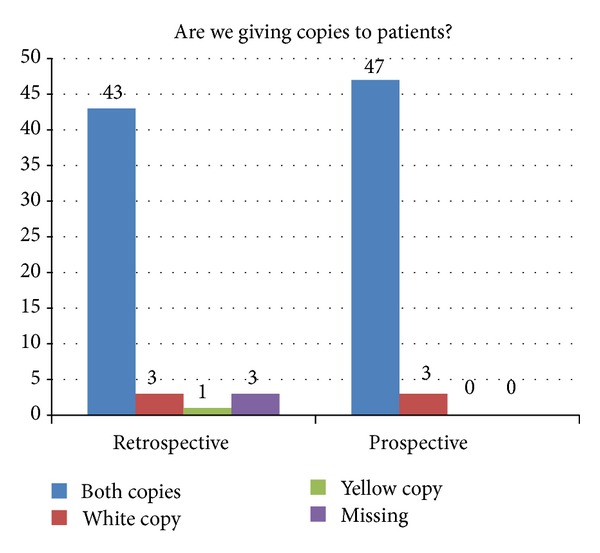
Allocation of consent copies to patient prior to surgery.

Only in 6% of cases (*n* = 3), in each phase, was the documentation of a copy of the consent form being given to the patient. The consent form used consisted of 2 sheets, with a carbon copy (white/case note's copy) attached to the handwritten front sheet (yellow/patient's copy) (Figure 2).

**Figure 3:**
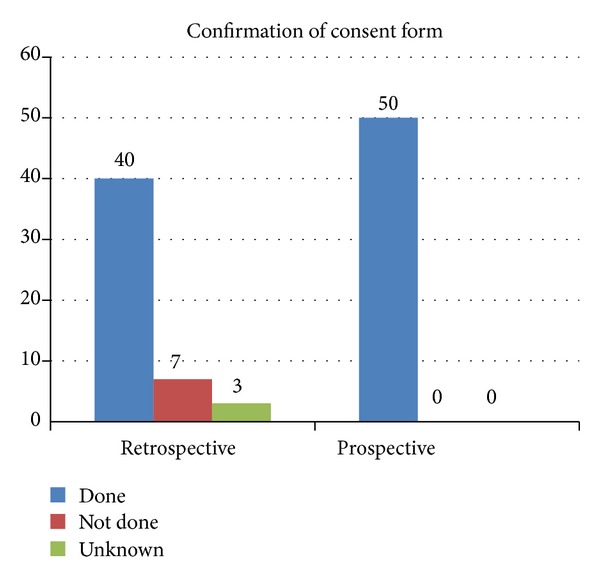
Confirmation of consent form by operating team in prior to operative procedure.

Another member of health care team confirmed consent prior to all cases in Phase 2, where as it was done in 80% (*n* = 40) cases in Phase 1 (Figure 3).

We found that it was common practice for the senior doctors (registrars/residents, consultants) to obtain consent from patients before trauma surgery in our unit. However, 14% (*n* = 7) of consent forms in Phase 1 and 22% of (*n* = 11) in Phase 2 were signed by Core Surgical Trainees year 2 who had been trained at departmental induction. British Orthopaedic Association (BOA) orthoconsent guideline was utilized as training reference for Junior Orthopaedic and Surgical Trainees [9].

The requirement for blood transfusion was discussed and recorded in 40% (*n* = 20) of cases where relevant in the first phase and 100% in Phase 2. Consent forms were completed thoroughly with respect to patient identifiers, legibility, responsible consultant's name, type of procedure, discussed risks and benefits, type of anaesthesia, and additional procedures like urinary catheterization in Phase 2 compared to incomplete data capture in Phase 1 (Table 1).

## 4. Discussion

This study demonstrates the need for training and awareness in consenting surgical patients amongst the medical staff. It was perceived that consenting practice was not thoroughly adhered to approved recommended guidelines during Phase 1. However, we managed to detect the drawbacks in our practice, discussed the results, and undertook adequate training of staff. The result was significantly improved in all aspects of consenting trauma patients during Phase 2 of the study. This is a clear example of how an audit cycle helped our unit to improve the standard of care as is expected in modern NHS culture.

This audit has demonstrated that by regular review of our practice and dissemination of the results service improvement can be achieved in a short time without involving extensive resources and increasing cost. The data in Phases 1 and 2 were blinded and hence the improvement observed was genuine.

More junior doctors had completed the consent process in Phase 2. Twenty-two percent consent forms were filled by junior grades in Phase 2 compared to 14% in Phase 1. Due to the time difference between Phases 1 and 2, there were more experienced Junior doctors in the Orthopaedic Department. Reviewing the notes, most doctors attending the patients in Phase 1 were foundation year trainees who were not fully aware of the operative procedures undertaken in trauma patients. In Phase 2, the patients were attended in most cases by Core Surgical Trainees year 2 (CT2). These doctors had completed their surgical membership examinations and were trained regarding consenting trauma patients at departmental induction in compliance with British Orthopaedic Association Consenting Guideline (Orthoconsent) [9] as an approved reference. All completed consent forms were reviewed and confirmed by senior surgeon or allocated clinical supervisor. This finding had no implications on the improvement of consenting practice that was observed.

Abbreviations were more commonly observed in Phase 1. This included common operations or surgical sites which were frequently abbreviated rather than being spelt out properly, for example, DHS for dynamic compression screw or L for Left. This was in contrast to the introduction of the National Patient Safety Goal to improve communication and restrict the use of abbreviations [10]. Abbreviations are commonly used in medical or surgical world to save time and space whilst writing in the patients' medical records [10]. This was noticed in 20% of consent forms irrespective of the grade and training. This is not certainly negligible and has been reported by other authors [7, 11]. This was addressed by focused training, communication, and feedback resulting in a significant decrease (*P* = 0.03) [Mann-Whitney *U*] in Phase 2.

All information regarding procedure-based benefits and relevant risks and/or alternatives had been documented using plain language.

The central notion of informed consent is that the patients have the proposed procedure explained to them in such a way that each can decide to proceed with the planned treatment [12]. In compliance with Department of Health “A guidance to health professional how to consent” the completed form should act and facilitate as an aid-memoire to caregivers and patients. A checklist of this kind provides adequate information and enables them to have a written record of the main points discussed based on agreement [3]. The allocation of the patient copy of the form must be recorded. This will help prevent future misunderstandings if a patient was to dispute the information that had been given.

Further improvement is essentially required in dispensing the copies and relevant procedure-based informative leaflets to patients to ensure that the patient receives at least very basic information about their treatment process including type of treatment, risks and benefits, type of anaesthesia, need of blood transfusion, and extraprocedures as required. Addressing these requirement is a fundamental part of seeking consent process [3, 4].

The consent form should be improved further to record the two-way flow of information. Suggestions include recording whether the patient had further questions, and whether the health care professional had fully read the notes of the patient/was completely conversant with the patient's problems. This topic could be looked into with the next audit cycle.

The limitations of this study are inherent to its nature. It is an audit of a small group of patients with a limited scope of service evaluation and improvement. Furthermore, similar discrepancies might exist in other areas of the trauma & orthopaedic deportment that were not evaluated in our present study.

However, the main purpose of this paper is to emphasize the need for regular service evaluation and effective communication of the data to improve patient care.

## 5. Conclusion

We recommended developing specific training sessions for junior doctors at a specific induction session on consenting common trauma procedures. When juniors are delegated to take consent, the operating surgeon should review the quality of the data recorded and still review the patient prior to surgery.

The variability of procedure-based risks or complications is vast and, therefore, we suggest the use of available online orthopaedic-basis procedure guideline (Orthoconsent) which has been endorsed and updated by the British Orthopaedic Association [9, 13] to guide and train the junior surgical or orthopaedic trainees to achieve competency in consenting trauma cases when is or are allocated to perform the procedure(s). These information sheets should be reviewed by each department locally, and relevant alterations made.

## Figures and Tables

**Table 1:** The results of completing consent forms according to established guidelines in two phases of the study. All data are presented as number of patients with the percentage in the parenthesis.

Data collected	Retrospective (Phase 1)	Prospective (Phase 2)
Use of abbreviations	37 (74%)	50 (100%)
Handing copies to patients	43 (86%)	47 (94%)
Confirmation of consent	40 (80%)	50 (100%)
Recording consultant	33 (66%)	50 (100%)
Patient's details	32 (64%)	50 (100%)
Consent for anaesthesia	37 (74%)	34 (68%)
Consent for blood transfusion	20 (40%)	50 (100%)
Proper procedure documented	47 (94%)	50 (100%)
Adequate benefits documented	46 (92%)	50 (100%)
Adequate risks documented	46 (92%)	50 (100%)
Additional procedures	6 (12%)	50 (100%)
